# Prevalence and risk factors for myopia in older adult east Chinese population

**DOI:** 10.1186/s12886-017-0574-4

**Published:** 2017-10-13

**Authors:** Cailian Xu, Chenwei Pan, Chunhua Zhao, Mingchao Bi, Qinghua Ma, Jianhui Cheng, E. Song

**Affiliations:** 1 The First Hospital of Jilin University, NO.71 Xinmin Street, Changchun, China; 2 Lixiang Eye Hospital of Soochow University, NO.200 Ganjiang Eastern Road, Suzhou, China; 3 Soochow University, Suzhou, China; 4 The 3rd People’s Hospital of Xiangcheng District, Suzhou, China

**Keywords:** Myopia, Risk factors, Prevalence, Sleep duration, Old adult

## Abstract

**Background:**

To determine the prevalence and associated factors for myopia and high myopia among older population in a rural community in Eastern China.

**Methods:**

A community-based, cross-sectional survey was conducted in the Weitang town located in Suzhou, an urban metropolis in East China. A total of 5613 Chinese residents aged 60 years and older were invited to complete a questionnaire and participated in a detailed eye examination,including measurements of visual acuity and refractive error using autorefraction and subjective refraction. Myopia and high myopia was defined as SE < −0.5 diopters (D) and < −5.0 D, respectively.

**Results:**

Among the 5613 participating individuals, 4795 (85.4%) complete refraction data of phakic right eye was included for analysis. The age-adjusted prevalence was 21.1% (95% confidence interval [CI], 19.9-22.2) for myopia and 2.5% (95% CI, 2.1-2.9) for high myopia. The prevalence of myopia tended to increase significantly with age(*p* < 0.001),and women had a higher rate of myopia than men (*p* < 0.001). According to multivariate logistic regression analysis, adults who were older (odds ration[OR]:1.05; 95% CI:1.04-1.07), spent more time for sleeping at night (OR:1.12;95% CI: 1.06-1.18),or had cataract (OR:1.60;95% CI:1.36-1.88) and family history of myopia (OR:1.47;95% CI:1.23-1.77), are more susceptible to myopia (p < 0.001). People who had older age, family history, cataract and specially longer night-time sleep duration, would have a higher risk of myopia.

**Conclusion:**

Myopia and high myopia among rural old adult population in Eastern China presents common. The current literature unanticipated suggests that there was a positive significant association between prevalence of myopia and night-time sleep duration among adult. Our data provide some evidence of this relationship and highlight the need for larger studies to further investigate this relationship longitudinally and explore mechanism therein.

**Electronic supplementary material:**

The online version of this article (10.1186/s12886-017-0574-4) contains supplementary material, which is available to authorized users.

## Background

Refractive error especially myopia is increasingly recognized as a significant cause of visual impairment and blindness globally [1, 2], and it brings a large socioeconomic burden on correction and treatment for complications of refractive error [3, 4]. The majority of related epidemiological studies ever reported focus on children and teenagers population, presenting myopia prevalence as high as 50% in Taiwan [5], 67.3% in Chinese mainland [6], 70% in Singapore [7], and even 96.5% in Korea [8]. As International Population Reports [9] reported that the number of people aged over 65 years is nearly 0.8 billion (11.0%) in 2010 and estimated would increase to 2.0 billion (22.0%) in 2050, the increasingly aging of the population has been more worthwhile to cause for concern. Thus, more research on refractive error which affects the quality of life by affecting vision, should be conducted in older adult population. Numerous epidemiological studies on refractive error are available for the older adult populations in European [10–13] and some Asian countries [14–16, 17], but in respect of mainland China only a few investigations [18–20] limit to hinder comprehensive cognition epidemiological characteristics of myopia, which were completed based a large adult resident population around several cities distributing concentrated in the north and southern China. Therefore, we conducted the present study in order to estimate the prevalence and associated risk factors of myopia among older adult population aged 60 and above in Eastern China.

## Methods

### Study population

Our study of refractive errors was a community-based, cross-sectional survey of eye diseases in China. It was part of the Weitang Geriatric Diseases Study that ever reported by our collaborative research group [21]. The Weitang Geriatric Diseases Study conducted in the Weitang town located in Suzhou, an urban metropolis in East China, was aimed to assess physiological and psychological state of Chinese older adult population and related factors. Accoding to the official records, there are 6030 older adults aged 60 years and over in the Weitang town. The study was approved by Soochow university ethics committee, and it was conducted according to the tenets of the Declaration of Helsinki of the World Medical Association regarding scientific research on human subjects.

### Interview and data collection

All eligible residents in the town were invited to the 3rd People’s Hospital of Xiangcheng District participating in the study, by sending a letter with the study objectives explanation to their each family. Besides, it was assured that the information collected would be strictly confidential. The questionnaires were administered by trained interviewers. A pre-designed and scientifically validated questionnaire (Additional file 1) was used to collect information on related factors of systemic diseases and personal habits, such as education level, occupation, marital status, income, housing, outdoor activity (yes/ no), sleep quality, and the number of hours per day for nightly sleep, sun exposure, watching TV and computer respectively. And we also interviewed a detailed medical history including smoking (current/past/never), alcohol consumption (yes/no), tea drinking, and whether the participant had previously been diagnosed diabetes mellitus or hypertension. Finally, we asked whether family members (within tertiary relatives) previously diagnosed with myopia.

### Clinical examination and definition

After interview, these participants ware carried out a detailed ophthalmologic examination in the 3rd People’s Hospital of Xiangcheng District, including autorefraction, visual acuity assessment, subjective refraction, slit lamp examination, fundus examination, and fundus photography. All participants underwent initial objective autorefraction with an autorefractometer (Canon RK-5 Auto Ref- Keratometer, Canon Inc. Ltd., Tokyo, Japan). The result of autorefraction was used as a starting point for a subsequent subjective refraction. Visual acuity was measured by trained research optometrists using a retro-illuminated Snellen chart with tumbling-E optotypes (Precision Vision, La Salle, IL, USA) at a distance of 4 m, and it was described in detail reported by other partial research of the Weitang Geriatric Diseases Study [22]. Because of the age of the study population, cycloplegia was not used. If the uncorrected VA was worse than 1.0, the refractive correction was carried out beginning with the results of autorefractometry, and the corrective lenses were adjusted manually to refine vision. Refraction data on all subjects were determined as follows. Initial objective refraction result was recorded if the uncorrected VA was 1.0 or better and manual subjective refraction was recorded if the uncorrected VA was worse than 1.0.

The spherical equivalent (SE; sphere +1/2 cylinder) was used for calculations of refractive error. In order to compare with other reports [23], myopia in our study was defined as SE < −0.5D,and high myopia SE < −5.0D, and also additionally defined as an SE of less than −0.75 or −1.0 D, and high myopia as an SE of less than −6.0*D. lens* opacity was graded using slit lamp microscope with the modified Lens Opacity Classification System (LOCS) III [24]. Cataract was defined as any score of nuclear, corneal or subcapsular opacity was beyond the LOCSIII standard. Fundus examination and photography was carried out to diagnose fundus lesions. On basis of physical examination, hypertension was defined as the measured systolic blood pressure ≥ 140 mmHg or the measured diastolic blood pressure ≥ 90 mmHg or ever diagnosed. Diabetes mellitus was defined as fasting plasma glucose ≥7.0 mmol/L or ever diagnosed.

### Statistical analysis

Participants with prior cataract surgery or incomplete documents were excluded from the analyses related to refractive error. As the Spearman correlation coefficients for SE (*r* = 0.76, *p <* 0.01) in the left and right eye were high, only right eye data were presented and analysed. Statistical analyses were performed using the statistical software Statistical Package for Social Science (SPSS ver.18.0; SPSS Inc., Chicago, ILUSA). The results were expressed as mean values ± standard deviation (SD) if the variables were continuous, or as percentage if categorical. Both univariate and multivariate logistic regression analyses were performed to study the effect of various factors on myopia and high myopia. From the univariate analysis, variables with *P* values <0.05 and those that were already established as risk factors were included in the multivariate logistic regression analysis. Two-tailed *P* < 0.05 was considered statistically significant. Odds ratios (OR) and 95% confidence intervals (95% CI) were shown.

## Results

A total of 5613 subjects was eligible to participate in the study from August 2014 to January 2015. Ultimately, 4611 subjects volunteered to participate in the hospital, and 1002 subjects did not attend the clinic then were examined at home. Excluded incomplete documents or subjects ever had a Lens surgery, there are 4795 (84.8%) refraction data of phakic rigt eye was included. The age ranged from 60 to 93 years with a mean of 67.5 ± 6.4 years, while 2331(48.6%) were male and 2464 (51.4%) were female. The mean ages of male and female were 67.4 ± 6.1 and 67.6 ± 6.5 years. Accordingly, there was no significant difference in age distribution between male and female (*p = 0.41*).

**Fig. 1:**
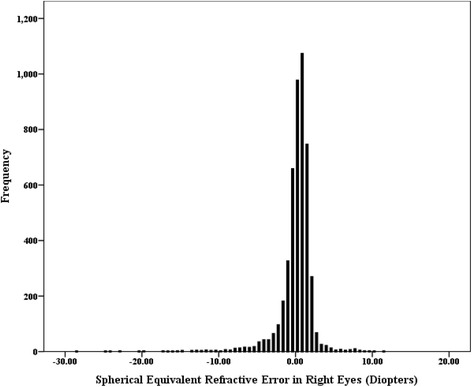
Distribution of spherical equivalent in the right eyes in elderly Chinese

Figure 1 presents the distribution of spherical equivalent in the elderly Chinese population. Prevalence of myopia and high myopia distributed by age and sex is shown in Table 1. After adjusted for age according to the 2010 population census of the People’s Republic of China, the age-standardized prevalence of myopia and high myopia with different definitions was 23.5% (SE *<* −0.5 D), 19.7% (SE *<* −0.75 D), 16.5% (SE *<* −1.0 D), 2.7% (SE *<* −5.0 D), and 2.1% (SE *<* −6.0 D), while the crude prevalence was 21.1%,17.2%,14.2%,2.5% and 2.0%, respectively. The prevalence of myopia (SE *<* −0.5 D) in female (24.2%) was higher than male (17.8%), and the gender difference was statistically significant (*p <* 0.001). The prevalence of high myopia (SE *<* −5.0 D) in female (3.0%) and male (2.0%) was presented with the same regularity. It was shown that the rate of myopia was increased significantly with age (*p <* 0.001), which was 15.8%, 16.7%, 22.8%, 37.9% and 43.5%, respectively in age groups of 60-64, 65-69, 70-74,75-79 and 80 years or older. Prevalence of myopia and high myopia in our study is compared with other studies in Table 2. Prevalence of myopia in our study was higher than the surveys reported based on Taiwan [25] and Harbin [20] (northeast china) residents, while was less than that in Guangzhou [26] (south china), Hon Kong [27], and Singaporean Chinese [23]. It approximated the result in Beijing Eye Study [18] and Handan Eye Study [19], which were conducted in North Chinese. On account of special data in some papers unavailable to us, the current result limited to compare roughly with studies among Chinese race older adult.

**Table 1:** Prevalence of Myopia and High Myopia in Chinese over 60 years old

		Myopia (SE < −0.5D)	Myopia (SE < −0.75D)	Myopia (SE < −1.0D)	Myopia (SE < −5.0D)	Myopia (SE < −6.0D)
	N (%)	n(%, 95% CI)	n(%, 95% CI)	n(%, 95% CI)	n(%, 95% CI)	n(%, 95% CI)
Total	4795	1010	827	683	121	97
Crude rate	–	21.1, 19.9-22.2	17.2, 16.2-18.3	14.2, 13.3-15.2	2.5, 2.1-2.9	2.0, 1.6-2.4
Age-standard rate^a^	–	23.5, 22.3-24.7	19.7, 18.6-20.8	16.5, 15.4-17.5	2.7, 2.2-3.1	2.1, 1.7-2.5
Gender						
Male	2331	414	336	282	46	36
	48.6	17.8, 16.2-19.3	14.4, 13.0-15.8	12.1, 11.8-13.4	2.0, 1.4-2.5	1.5, 1.0-2.0
Female	2464	596	491	401	75	61
	51.4	24.2, 22.5-25.9	19.9, 18.4-21.5	16.3, 14.8-17.7	3.0, 2.4-3.7	2.5, 1.9-3.1
Age group,y						
60-64	1950	308	228	179	37	32
	40.7	15.8, 14.2-17.4	11.7, 10.3-13.1	9.2, 7.9-10.5	1.9, 1.3-2.5	1.6, 1.1-2.2
65-69	1364	230	118	144	31	28
	28.4	16.7, 14.9-18.9	13.8, 12.0-15.6	10.6, 8.9-12.2	2.3, 1.5-3.1	2.1, 1.3-2.8
70-74	698	159	136	120	25	17
	14.6	22.8, 19.7-25.9	19.5, 16.5-22.4	17.2, 14.4-20.0	3.6, 2.2-5.0	2.4, 1.3-3.6
75-79	493	187	159	139	18	16
	10.3	37.9, 33.6-42.2	32.3, 18.1-36.4	28.2, 24.2-32.2	3.5, 2.0-5.3	3.2, 1.7-4.8
80 and older	290	126	116	101	10	4
	6.0	43.5, 37.7-49.2	40.0, 34.3-45.7	34.8, 29.3-40.3	3.4, 1.3-5.6	1.4, 0.0-2.7

*SE* spherical eqivalent, *D* diopter, *CI* confidence interval

^a^Age-standardized to the 2010 population census of the People’s Republic of China

**Table 2:** Different Prevalence of Myopia in Partial Population-Based Studies for Chinese Race in Elderly Population

Study	Age groups, (%)			
	60-69	70-79	80 +	all
Myopia (SE < −0.5D)				
Suzhou(Our study)	16.2	29.1	43.5	21.1
Guangzhou [27]	31.1	31.3	46.7	32.6
Handan [21]	14.4	34.3	42.9	21.4
Beijing [20]	NA^a^	NA	NA	21.8
Harbin [22]	11.3	12.8		9.5
Taiwan [26]	12.8	21.6	23.3	18.3
Singaporean Chinese [29]	30	27		35.0
Hon Kong [28]	NA	NA	NA	41.1
High Myopia (SE < −5.0D)				
Suzhou(Our study)	2.1	3.6	3.4	2.5
Guangzhou [27]	6.6	3.4	1.3	4.7
Handan [21]	2.4	4.5	6.1	3.1
Beijing [20]	NA	NA	NA	3.3
Harbin [22]	NA	NA	NA	1.4
Taiwan [26]^b^	1.9	2.6	1.4	2.3
Singaporean Chinese [29]	NA	NA	NA	8.2
Hon Kong [28]	6.6	4.4		6.9

^a^NA: Data was not available

^b^High myopia was defined as SE < −6.0D

**Fig. 2:**
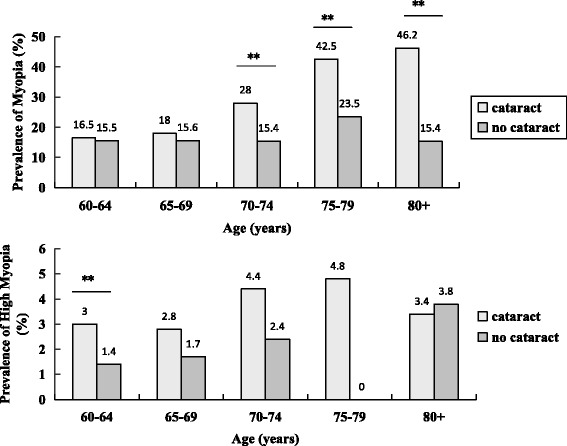
Prevalence of myopia and high myopia by age for subjects with or without cataract respectively. (*p < 0.01, **p < 0.001)

Based on LOCS III, there were 2360 vs 2435 (50.8%:49.2%) subjects with or without cataract respectively. Figure 2 shows the prevalence of myopia and high myopia in different age groups respectively with cataract or not. The myopia rate of cataract group increased with age, and there was similar trend in subjects without cataract from 60 to 80 years group, but decreased in followed 80+ years group. The myopia rate of cataract subjects (26.4%) was statistically significantly higher than group without cataract(15.9%) (*p <* 0.001). In older groups with 70 year and above but not youngers, myopia prevalence is statistically significant between cataract subjects and others (chi-square test, *p* = 0.55, *p* = 0.25, *p <* 0.001, *p <* 0.001, *p* = 0.003 respectively).

Because relative lack of sample capacity of older subject with high myopia, the high myopia rate in the population results differently that *p* value less than 0.05 only appears in 60-64 age group.

**Fig. 3:**
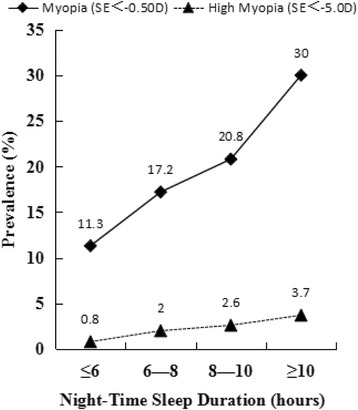
Line graphs of prevalence of myopia and high myopia by night-time sleep duration

Individual factors such as age, gender, education, occupation, income, marital status, housing area, smoking, alcohol consumption, tea drinking, time for sleeping per night, outdoor activities, family history of myopia and cataract, were statistically significant (*p < 0.05*) related with myopia shown in Table 3. With multivariate logistic regression analysis, various models were constructed and selectively presented in Table 4. Those who spent more time for sleeping at night (OR = 1.12), were with older age (OR = 1.05),or had cataract (OR = 1.60) and family history of myopia (OR = 1.47), are more susceptible to myopia (*p <* 0.001). Night-time sleep duration (9.1 ± 1.5 h) in myopes was significantly high than non-myopes (8.6 ± 1.4 h) (F = 85.6,*p <* 0.001*)*. Prevalence of myopia as well as high myopia increased with night-time sleep duration. Figure 3 was shown prevalence of myopia and high myopia both increased with night-time sleep duration. And there was statistical significance between adults with and without myopia (*p <* 0.001), and also in high myopia (*p =* 0.008).

**Table 3:** Single variable Logistic Regression Analysis of the risk factors associated with Myopia and High Myopia

	Myopia (SE < −0.5D)			High Myopia (SE < −5.0D)		
	Beta	OR (95% CI)	*p*	Beta	OR (95% CI)	*p*
Age, y	0.07	1.08 (1.07-1.09)	**<0.001**	0.04	1.04 (1.01-1.07)	**0.004**
Gender						
Male	0	1		0	1	
Female	0.39	1.48 (1.28-1.70)	**<0.001**	0.44	1.56 (1.08-2.26)	0.12
Educational Level						
Illiterate or no education	0	1		0	1	
Primary education	−0.45	0.64 (0.55-0.75)	**<0.001**	−0.23	0.79 (0.54-1.17)	0.24
Secondary education and above	−0.46	0.63 (0.51-0.79)	**<0.001**	0.64	0.51 (0.26-1.01)	0.05
Occupation						
Peasants	0	1		0	1	
Workers	−0.40	0.67 (0.57-0.79)	**<0.001**	−0.83	0.44 (0.26-0.72)	**0.001**
Professionals	−0.83	0.44 (0.28-0.69)	**<0.001**	−0.10	0.90 (0.36-2.26)	0.83
Managers	−0.84	0.43 (0.20-0.91)	**0.03**	−0.73	0.48 (0.07-3.53)	0.47
Teachers	−0.23	0.80 (0.42-1.51)	0.48	1.27	3.56 (1.49-8.51)	**0.004**
Civil servants	0.13	1.13 (0.36-3.57)	0.83	−19.12	–	1.00
unknown	−0.45	0.64 (0.49-0.83)	**0.001**	−0.52	0.60 (0.29-1.24)	0.12
Individual monthly income						
Less than ¥1000	0	1		0	1	
¥1001-4000	−0.36	0.70 (0.60-0.81)	**<0.001**	−0.21	0.81 (0.55-1.20)	0.29
More than ¥4000	−0.53	0.59 (0.38-0.92)	**0.020**	0.18	1.20 (0.48-3.01)	0.70
Marital status						
Married	0	1		0	1	
Single /Divorce /Widowed	0.42	1.53 (1.28-1.82)	**<0.001**	0.10	1.11 (0.69-1.79)	0.67
Housing area, m^2^						
Less than 80	0	1		0	1	
80-130	−0.40	0.67 (0.53-0.84)	**0.001**	−0.52	0.59 (0.32-1.11)	0.10
More than 130	−0.31	0.73 (0.62-0.86)	**<0.001**	−0.26	0.77 (0.51-1.16)	0.21
Smoking						
Never	0	1		0	1	
Past	−0.32	0.73 (0.56-0.95)	**0.02**	−0.15	0.86 (0.44-1.67)	0.66
Current	−0.36	0.70 (0.59-0.82)	**<0.001**	−0.39	0.68 (0.43-1.07)	0.10
Alcohol consumption						
No	0	1		0	1	
Yes	−0.49	0.62 (0.51-0.74)	**<0.001**	−0.25	0.78 (0.50-1.24)	0.30
Tea drinking						
No	0	1		0	1	
Yes	−0.42	0.66 (0.56-0.77)	**<0.001**	−0.38	0.69 (0.46-1.03)	0.07
Time for sleeping per night,h	0.22	1.25 (1.19-1.31)	**<0.001**	0.19	1.21 (1.08-1.36)	**0.002**
Sleep quality						
Well	0	1		0	1	
Poor	0.08	1.08 (0.85-1.37)	0.52	0.33	1.40 (0.81-2.42)	0.23
Time for watching television per day,h	−0.17	0.84 (0.80-0.89)	**<0.001**	−0.30	0.74 (0.63-0.87)	**<0.001**
Time for playing computer per day,h	−0.07	0.93 (0.76-1.14)	0.50	−0.04	0.96 (0.58-1.61)	0.89
Time for outdoor activities per day,h	−0.03	0.97 (0.94-1.01)	0.12	0.002	1.00 (0.91-1.10)	0.97
Vegetarian	0.14	1.15 (0.67-1.95)	0.62	0.03	1.03 (0.25-4.25)	0.97
Family history of myopia	0.26	1.29 (1.09-1.53)	**0.003**	0.14	1.15 (0.74-1.80)	0.54
Hypertension	0.16	1.18 (1.00-1.38)	0.05	0.02	1.02 (0.68-1.55)	0.91
Diabetes	−0.13	0.88 (0.70-1.10)	0.25	−0.39	0.68 (0.35-1.31)	0.25
Cataract	0.64	1.90 (1.65-2.19)	**<0.001**	0.83	2.30 (1.56-3.39)	**<0.001**

*OR* odds ratio, *CI* confidence interval. The significant values (*p* < 0.05) are presented in bold

**Table 4:** Multivariate Logistic Regression Analysis of the risk factors associated with Myopia and High Myopia

	Myopia (SE < −0.5D)			High Myopia (SE < −5.0D)		
	B	OR (95% CI)	*p*	B	OR (95% CI)	*p*
Age, y	0.05	1.05 (1.04-1.07)	**<0.001**	0.00	1.00 (0.97-1.04)	0.86
Gender						
Male	0	1		0	1	
Female	0.21	1.26 (0.98-1.56)	0.08	−0.41	0.67 (0.36-1.24)	0.20
Educational Level						
Illiterate or no education	0	1		0	1	
Primary education	−0.17	0.85 (0.71-1.00)	0.05	0.00	1.00 (0.65-1.55)	0.99
Secondary education and above	−0.19	0.82 (0.63-1.08)	0.15	1.02	2.77 (1.11-6.94)	**0.03**
Occupation						
Peasants	0	1		0	1	
Workers	−0.07	0.93 (0.78-1.12)	0.45	0.66	1.93 (1.12-3.31)	0.02
Professionals	−0.53	0.59 (0.36-0.95)	**0.03**	−0.30	0.74 (0.28-2.00)	0.56
Managers	−0.48	0.62 (0.29-1.34)	0.22	0.28	1.32 (0.18-10.02)	0.79
Teachers	−0.14	0.87 (0.42-1.79)	0.70	−2.03	0.13 (0.04-0.46)	**0.002**
Civil servants	0.35	1.42 (0.42-4.79)	0.57	18.32	–	0.999
unknown	−0.11	0.9 (0.68-1.19)	0.45	0.33	1.39 (0.65-2.96)	0.40
Individual monthly income						
Less than ¥1000	0	1		0	1	
¥1001-4000	0.14	1.15 (0.97-1.37)	0.11	−0.17	0.84 (0.54-1.31)	0.44
More than ¥4000	0.17	1.18 (0.72-1.95)	0.52	−0.31	0.73 (0.24-2.25)	0.59
Marital status						
Married	0	1		0	1	
Single /Divorce /Widowed	−0.08	0.92 (0.75-1.12)	0.41	0.26	1.29 (0.77-2.18)	0.34
Housing area, m^2^						
Less than 80	0	1		0	1	
80-130	−0.15	0.86 (0.67-1.09)	0.21	0.44	1.55 (0.81-2.95)	0.19
More than 130	−0.04	0.96 (0.81-1.14)	0.65	0.11	1.12 (0.73-1.73)	0.61
Smoking						
Never	0	1		0	1	
Past	0.19	1.21 (0.94-1.56)	0.13	−0.21	0.81 (0.41-1.61)	0.55
Current	0.09	1.09 (0.79-1.51)	0.58	−0.29	0.75 (0.33-1.69)	0.48
Alcohol consumption						
No	0	1		0	1	
Yes	−0.22	0.80 (0.65-1.00)	0.05	−0.17	0.84 (0.48-1.49)	0.55
Tea drinking						
No	0	1		0	1	
Yes	−0.19	0.83 (0.69-1.00)	0.05	0.28	1.32 (0.81-2.15)	0.27
Time for sleeping per night,h	0.11	1.12 (1.06-1.18)	**<0.001**	−0.16	0.85 (0.74-0.97)	**0.02**
Family history of myopia						
No	0	1		0	1	
Yes	0.39	1.47 (1.23-1.77)	**<0.001**	−0.33	0.72 (0.45-1.14)	0.16
Cataract						
No	0	1		0	1	
Yes	0.47	1.60 (1.36-1.88)	**<0.001**	−0.87	0.42 (0.27-0.65)	**<0.001**

*OR* odds ratio, *CI* confidence interval. The significant values (*p* < 0.05) are presented in bold

## Discussion

This survey provides the first population-based cross-sectional data on the prevalence of myopia and high myopia and associated risk factors among the older adult in East China. The major finding was that myopia is common in East Chinese resident population with age ≥ 60 years. It indicated that 21.1% subjects had myopia, which was much lower than the rate (41.8%) among Japanese urban population with age ≥ 65 reported in the Tajimi study [18]. However, it was higher than the rate among rural Korean population in the same age group (13.2% in 60-69 years, 15.9% in 70-79 years, and 34.9% in 80+ years) [29]. The prevalence of myopia among older adult population in East China was lower than South China, and approximated North China. Different distribution of myopia in Chinese cities may suggest that environment and lifestyle may play an important role in myopia among older population, except reasons such as differences in ages of subjects included and the examination techniques used in different studies. The prevalence of myopia in this elderly population was increased with age, which was similar reported in south Indian [30] and the black population [31], and also in Chinese population such as southern Harbin, the Handan Eye Study and the Liwan Eye Study. We noted that the myopia rate of cataract subjects in our study was higher than those without cataract and similarly increased with age. This pattern between myopia and age, possibly due to lens opacity especially nuclear one in elderly population, had been widely explained in other studies [13, 31]. People with high myopia are reported more susceptible to ocular complication such as cataract, glaucoma, retinal detachment [32], which directly or indirectly cause a burden of visual impairment. The prevalence of high myopia (defined as SE < −5.0D) in the present study was higher than the Harbin eye study, near to Taiwan as high myopia was defined as SE < −6.0D, and lower than the other mostly studies.

Through univariate and mutivariate logistic regression analysis in this population, we confirmed that significant risk factors associated with myopia were occupation,family history of myopia and time for sleeping per night, except age and cataract which was mentioned previously. Occupation as a myopic factor in our study, might be interrelated with education, which has been expounded clearly in other population based study [33]. It was discovered that risk of myopia increased with a positive family history, in agreement with the viewpoint that familial effects on the level and onset of myopia [34, 35], and genetic factors play importantly in myopic development [36].

In this study, we considerably discovered somewhat evidence of association between night-time sleep duration and myopia in the older Chinese population. Adults who spent more time for sleeping at night had a significantly higher prevalence of myopia. After searching for the words “sleep” and “myopia” /“refrative error” in PubMed up to June 2017, there was no related published epidemiological studies based on older population, therefore it is hard to accordingly confirm the association between sleep duration and myopia in adult. The articles focused on children and teenagers represent that mean night-time sleep duration was actually longer among myopic children than non-myopic ones in children, which was possibly resulted that sleep duration was longer in myopic children as a marker of disordered sleep [37]. The pattern between myopia and night-time sleep duration among Chinese older adult population in our study was consistent to children, potentially attributed to similar reasons. Although we cannot establish causality using such cross-sectional data, it is possibly explained that longer sleep duration in myopia is associated with decreased sleep quality [38], which generating from their somewhat worse physical condition such as cataract [39], visual impairment [40] or disability in action [41], et al. This category who use to have a prolonged night-time sleep, is likely to accompanied by myopia. However, our result was contrary to the study among the Korean adolescents which indicated inverse relationship between sleep duration and myopia [42]. Such inverse relationship was accounted possibly for chronic sleep deprivation in korean students, which occurred as a result of high educational intensity characterized by amounts of near work and little time outdoor activities. Compared with the study in Korean adolescents, key confounder including outdoor activity time and near work such as playing computer was measured in analysis, and ran insignificantly finally in current study [4]. Additionally, the association between outdoor activities and myopia occurred insignificantly in this study, that might possibly remind some other underlying modes for sleep duration affecting myopia in older adults, and it is in need of further related research especially about mechanisms.

Our study performed a epidemiological data on myopia in Chinese older adult population, but there are some limitations. First, myopia prevalence was estimated based on refraction data individually rather than biometric data especially axial length in union. Second, the majority of associated risk factors were obtained through interview questionnaires, therefore recall bias could not be avoided and tended to increase in older subjects. Finally, as the current study was cross-sectional, it was difficult to infer causal relationship between myopia and sleep, as well as other risk factors.

## Conclusions

The present study provides epidemiological data on myopia and high myopia in Chinese rural old adult population. The prevalence and risk factor of myopia represent not exactly the same in other races and other parts of China, which might show differences in environmental conditions, lifestyles as well as genetic reasons for association with refractive error. This article is the first to suggest the association between sleep duration and myopia in adults aged 60 years and over. Though we found somewhat evidence of a positive association between night-time sleep duration and myopia, only night-time sleep duration which self-reported were included in analysis of association between sleep and myopia. In order to clarify the detail association between sleep and myopia, more further research should be conducted in different age groups and ethnic population, especially focused on the mechanism, and also filled in regard to midday/(night-time + midday) sleep duration and quantitative index for sleep quality.

## Additional file

Additional file 1: Questionnaires. English language versions of all questionnaires used in this study. (DOC 18 kb)

## Abbreviations

CI: Confidence interval
D: Diopters
LOCS: Lens Opacity Classification System
OR: Odds ration
SD: Standard deviation
SE: Spherical equivalent
VA: Visual acuity
