# Supplementary material for: Prevalence and risk factors for myopia in older adult east Chinese population
Source: BMC Ophthalmol. 2017 Oct 13;17:191. doi: 10.1186/s12886-017-0574-4 (PMC5640960; doi:10.1186/s12886-017-0574-4)
Supplement: Additional file 1: — Questionnaires. English language versions of all questionnaires used in this study. (DOC 18 kb) [file 12886_2017_574_MOESM1_ESM.doc]

QUESTIONNAIRES

1. What is your educational level? “Illiterate or no education” or “Primary education” or “Secondary education and above”.
2. What is your current occupation? Peasants, workers, professionals, managers, teachers,civil servants or unknown.
3. How much is your monthly salary?
4. Did you married ? Married, single,divorce or widowed?
5. What's the area of housing?
6. Have you ever smoke every week for at least one year in your life?
7. Are you still smoking often now?
8. How many years have you smoke?
9. Have you ever drunk alcohol every week for at least one year in your life?
10. Are you still drinking alcohol often now?
11. How many years have you drunk alcohol?
12. Have you ever drunk tea every week for at least one year in your life?
13. Are you still drinking tea often now?
14. How many years have you drunk tea?
15. Who have myopia in your family? Nobody, father/mother, sister/brother, grandparents, son/daughter, grandchild.
16. Are you a vegetarian? Yes or no?
17. What time do you go to sleep usually?
18. What time do you get up usually?
19. What is your sleep quality? Well or poor?
20. How many hours do you spent for watching television per day?
21. How many hours do you spent for playing computer per day?
22. How many hours do you spent for outdoor activities per day?
